# PDZ-Containing Proteins Targeted by the ACE2 Receptor

**DOI:** 10.3390/v13112281

**Published:** 2021-11-15

**Authors:** Célia Caillet-Saguy, Nicolas Wolff

**Affiliations:** Unité Récepteurs-Canaux, Institut Pasteur, UMR CNRS 3571, 75015 Paris, France

**Keywords:** SARS-CoV-2, ACE2, host factors, binding assay, PDZ domain

## Abstract

Angiotensin-converting enzyme 2 (ACE2) is a main receptor for SARS-CoV-2 entry to the host cell. Indeed, the first step in viral entry is the binding of the viral trimeric spike (S) protein to ACE2. Abundantly present in human epithelial cells of many organs, ACE2 is also expressed in the human brain. ACE2 is a type I membrane protein with an extracellular N-terminal peptidase domain and a C-terminal collectrin-like domain that ends with a single transmembrane helix and an intracellular 44-residue segment. This C-terminal segment contains a PDZ-binding motif (PBM) targeting protein-interacting domains called PSD-95/Dlg/ZO-1 (PDZ). Here, we identified the human PDZ specificity profile of the ACE2 PBM using the high-throughput holdup assay and measuring the binding intensities of the PBM of ACE2 against the full human PDZome. We discovered 14 human PDZ binders of ACE2 showing significant binding with dissociation constants’ values ranging from 3 to 81 μM. NHERF, SHANK, and SNX27 proteins found in this study are involved in protein trafficking. The PDZ/PBM interactions with ACE2 could play a role in ACE2 internalization and recycling that could be of benefit for the virus entry. Interestingly, most of the ACE2 partners we identified are expressed in neuronal cells, such as SHANK and MAST families, and modifications of the interactions between ACE2 and these neuronal proteins may be involved in the neurological symptoms of COVID-19.

## 1. Introduction

Three human coronaviruses have appeared since the early 2000s and caused epidemics: SARS-CoV (2003), MERS-CoV (2012), and finally SARS-CoV-2 (2019). However, only SARS-CoV-2 has become a pandemic in a few months.

SARS-CoV-2 is a 30 kb RNA virus that belongs to the betacoronavirus genus and must enter a host cell in order to be able to replicate. The first step in this process is therefore the entry of viral material into the cytoplasm after passing through the cell membrane. The entry step begins with the attachment of the viral particle to the cell surface. This is based on the interaction between the spicules on the surface of the viral particle (spike protein S of SARS-CoV-2) and the glycoprotein angiotensin-converting enzyme 2 (ACE2) which acts as an entry receptor [1]. After attachment to ACE2, the viral protein S is cut into two parts (S1 and S2) by the transmembrane serine protease 2 (TMPRSS2), which is present on the surface of the host cell [2]. Then, furin cleaves the S2 region (S2′) to start conformational changes for fusion between the viral envelope and the plasma membrane of the host cell. Therefore, the “fusion peptide”, from residues 816 to 857 of protein S [3], is exposed for insertion into the host cell membrane. There follows a gathering between the envelope of the virus and the cell membrane, both formed by a lipid bilayer which will therefore fuse [4]. In a second ACE2-dependent route, the virus can also enter by endocytosis by the cathepsin-L pathway. Cathepsin L is ubiquitously expressed in mammalian cells and cleaves the S2′ and activates the fusion between viral and endosomal membranes, leading to the release of the viral RNA into the host cell cytoplasm [5] where the virus replicates [6].

The presence of the ACE2 viral receptor is a major determinant of the specific recognition between the virus and the host. SARS-CoV-2 can therefore infect human cells expressing ACE2. The expression profile of ACE2 is broad. The highest expression levels of ACE2 are found in the small intestine, testis, kidneys, heart, thyroid and adipose tissue; intermediate in the lungs, colon, liver, bladder and adrenal glands; and lowest in the blood, spleen, bone marrow, brain, blood vessels and muscle [7]. Symptomatic patients with SARS-CoV-2 infection are often reported as having fever, cough, nasal congestion, fatigue and other signs of an upper respiratory tract infection, which can develop into acute respiratory distress syndrome (ARDS) with a low survival rate [8]. Furthermore, ACE2 is essential to the regulation of the renin–angiotensin–aldosterone system (RAAS), which is a main regulator of blood pressure as well as fluid and electrolyte homeostasis.

**Figure 1:**
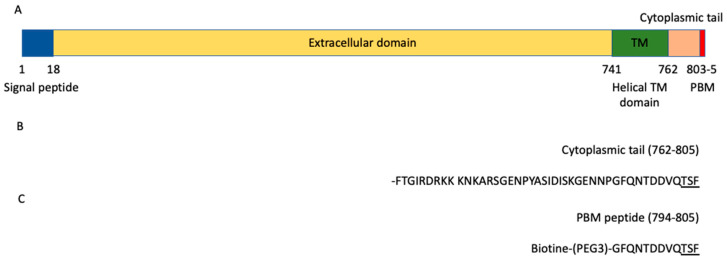
ACE2 domain organization. (**A**) Schematic domain organization of the ACE2 protein. (**B**) Sequence of the cytoplasmic tail (762–805) of ACE2. (**C**) Sequence of the peptide used in the holdup assay. The PBM is underlined in the sequences.

ACE2 is a glycosylated integral membrane protein of 805 residues with a large N-terminal ectodomain (residues 18–740), a transmembrane domain of 21 residues (residues 741–761) and a short cytoplasmic C-terminal region (residues 762–805) containing a PDZ (PSD-95/Dlg/ZO-1)-binding motif (PBM) at the C-terminal extremity (residues 803–805; sequence-TSF_COOH_) [9] (Figure 1A). This PBM of class I with the motif sequence (S/T)-X-Φ_COOH_, where X is any residue (position −1), and Φ is a hydrophobic residue (last position 0) (Figure 1B), is enabled to bind to other proteins through their PDZ domains. The affinities of a FITC-labeled C-terminal peptide of ACE2 796–805 were previously measured for eight PDZ domains by fluorescence polarization assays [10]. Three PDZ domains, the first PDZ domain (PDZ1) of NHERF3 and the PDZ domains of SHANK1 and SNX27, were found to bind to the ACE2 PBM peptide. However, only 8 of 272 PDZ expressed in the human proteome (the PDZome) were tested in this study. Recently, ACE2 was also shown to interact with NHERF1 PDZ domains enhancing the ACE2 membrane residence, and consequently increasing the ACE2-mediated SARS-CoV2 cell entry [11].

The PBM–PDZ interactions are involved in a wide variety of critical cellular processes and are often disturbed in pathologies and by viral proteins during infection. The human proteome contains 272 PDZ domains distributed over 152 proteins, as demonstrated in Luck et al. [12], but may vary depending on databases and identification methods [13]. Recently, we used the holdup assay [14] adapted to low-to-medium (1–100 μM) affinity, for high-throughput quantification of PDZ–PBM interaction to screen the PBMs of SARS-CoV-2 E, 3a and N proteins against the PDZome [15].

In this study, we used the holdup assay to identify the cellular PDZ-containing proteins targeted by ACE2 through a PDZ–PBM interaction among the full PDZome. We established a list of PDZ-containing partners potentially involved in ACE2 cell signaling through its PBM. We identified 14 PDZs that significantly bind to the ACE2 PBM and determined their affinities. Interestingly, the NHERF, SHANK and SNX27 proteins found in this study could play a role in ACE2 internalization and recycling that could benefit the virus entry. In addition, the ACE2 PDZ-binding motif binds notably to neuronal proteins, SHANK and MAST1/MAST2 proteins, that could participate in the neurological alterations in patients infected by SARS-CoV-2.

## 2. Materials and Methods

### 2.1. Peptide Synthesis

The biotinylated peptide (sequence Biotin-(PEG3)-GFQNTDDVQTSF) (Figure 1C) was synthesized in solid phase using Fmoc strategy (Proteogenix). The sequence encompasses a biotinyl group, a (PEG, polyethylene glycol)_3_ spacer and the C-terminal sequence of the human ACE2 protein (UniProtKB-Q9BYF1) corresponding to the last twelve residues. Peptides were resuspended in the buffer used for holdup.

### 2.2. Holdup Assay

The human PDZ domains library was produced according to the high-throughput protocol previously described [16]. Briefly, after expression of the His6–MBP–PDZ constructs in *Escherichia coli* and lysis of bacteria, the constructs were adjusted to a concentration of about 4 μM in the soluble fractions of the cell lysate and frozen in 96-well plates.

The holdup assay was carried out as previously described [14,16]. We measured the interactions of the peptide against the complete human PDZome containing 266 PDZ domains out of 272 PDZ domains identified in the human genome [13]. Error bars are the standard deviations of two independent experiments. Briefly, cell lysates containing PDZ were incubated with streptavidin resin saturated with biotinylated PBM peptides or with biotin used as a negative control. The supernatant was separated from the resin by a rapid filtration step using filter plates and then the concentrations of PDZ in the supernatant were measured using the microcapillary electrophoretic system (Caliper; PerkinElmer, Waltham, MA, USA).

Binding intensity (BI) values were calculated using the formula: BI = (Itot − Idepl)/Itot, where Itot is the total peak intensity of PDZ (biotin control) and Idepl is the intensity of the PDZ peak in the presence of the peptide. BI = 0.2 was used as the minimum BI cutoff value to define high confidence PDZ–PBM pairs, as previously proposed [14]. For detailed protocols, see references [14,16].

### 2.3. Conversion of BI Values to Dissociation Equilibrium Constants

**Figure 2:**
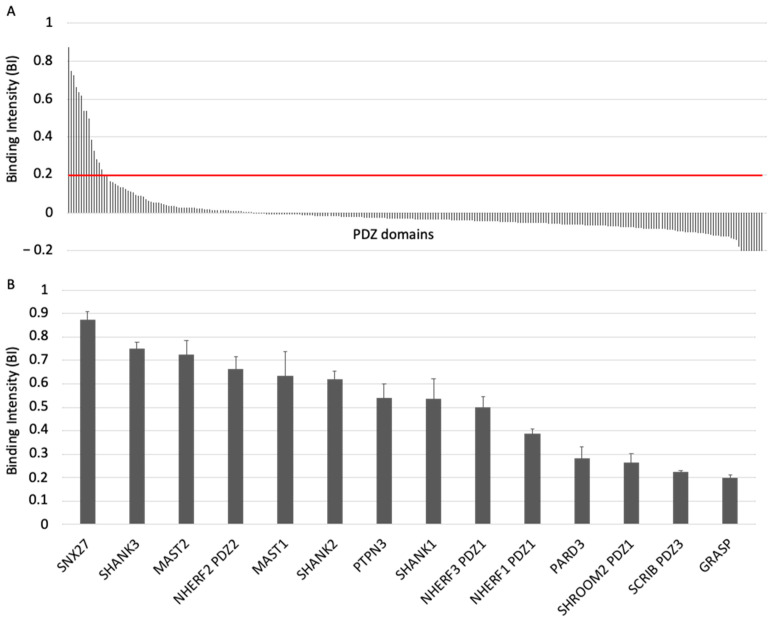
PDZome-binding profile of ACE2 protein PBM by holdup assay. (**A**) PDZ domains are ranked by decreased BI values. A red line indicates the threshold of significant BI at 0.2. (**B**) Zoomed-in view shows the 14 PDZ binders of ACE2 PBM peptide, which displayed BI values higher than 0.2. The data are representative of two independent experiments and error bars correspond to the standard deviation.

Dissociation constants (Kd) values were calculated from the BIs (Figure 2, Table S1) according to the method published in Gogl et al. [17].

BIs were transformed into dissociation constants (KD) using the following formula:

Kd = (([PDZtot] − BI × [PDZtot]) × ([PBMtot] − BI × [PDZtot]))/(BI × [PDZtot])(1)

where [PDZtot] and [PBMtot] correspond to the total concentrations of the PDZ domain (typically 4 μM) and the PBM peptide, respectively, used during the assay. The PBMtot concentration in the resin during the holdup assay may differ from one peptide to another. Thus, to convert BI values into Kd constants, we used the Kd values of the three PBM–PDZ interactions previously reported between the ACE2 PBM peptide and three PDZ domains (PDZ1 NHERF3, and the PDZ domains of SHANK1 and SNX27) measured by fluorescence polarization assays [10]. These affinities were used to back-calculate the PBM peptide concentrations in the holdup assays. We found that all determined BI–Kd pairs resulted in a mean immobilized peptide concentration of 21 μM, a concentration that is coherent with other peptides in the same method [15,17,18]. The minimal BI threshold value of 0.2, defined for significant detectable binders, roughly corresponds to an 80 μM Kd value, as previously reported [15].

## 3. Results

### 3.1. The ACE2 PDZ-Binding Motif Recognizes 14 PDZ Domains with Affinity Values of Interactions Ranging from 3 μM to 81 μM

To investigate the specificity profile of the PBM of ACE2 against all human PDZ domains, we used the automated high-throughput holdup assay, which allows measuring binding intensities (BIs) for a large number of PDZ domain–PBM pairs [14]. We used an updated PDZome library that contains 266 human PDZ domains [16]. The ACE2 PBM peptide was synthetized as a 12-mer encompassing the C-terminal PBM sequence of ACE2 protein linked to an N-terminal biotinyl group (Figure 1C). The PDZome-binding profile (Figure 2A) represents the individual BIs of each PDZ domain for the ACE2 PBM peptide. The binding intensities are directly linked to the PBM/PDZ affinities. Thus, the holdup approach shows a high sensitivity to detect low-to-medium affinity PDZ/PBM pairs. It provides an affinity-based ranking of the identified binders corresponding to a specificity profile (Figure 2A). Based on BI values higher than 0.2 for significant PDZ–peptide interactions [14], 14 PDZ domains were identified (Figure 2B, Table S1). This dataset represents about 5% of the human PDZome targeted by the PBM of ACE2, as illustrated by the sharp profile of interaction of Figure 2A, reflecting the specificity of these interactions.

We converted the holdup BI values to Kd values, as described previously [17,18]. Using the minimal threshold BI value of 0.2, corresponding to a Kd value of ~80 μM in this study, the fourteen PDZ binders of ACE2 PBM showed interactions with affinities values ranging from 3 μM (SNX27) to 81 μM (general receptor for phosphoinositides 1-associated scaffold protein, also named Tamalin (GRASP)) (Figure 2B, Table S1). Thus, the canonical PBM sequence of ACE2 protein binds *in vitro* to several PDZ domains in the 1–100 μM affinity range typically found for PDZ/PBM interactions. The PDZ of SNX27 is the best binder of ACE2 PBM in our assay, while three other PDZs present Kd values in the micromolar range: the PDZ domains of SHANK3 and of MAST2, and PDZ2 of NHERF2 (Table S1). These are considered as strong interactions for PDZ/PBM complexes. Then, the PDZ domains of MAST1, SHANK1, SHANK2, PTPN3, PARD3, GRASP, PDZ1 of NHERF3 and NHERF1 of SHROOM2 and the PDZ3 of SCRIB have intermediate Kd values between 10 and 81 μM for the ACE2 PBM peptide.

### 3.2. The PDZ-Binding Motif of ACE2 Targets Proteins Involved in Protein Trafficking and Some Proteins Preferentially Expressed in Neurons

We determined a list of PDZ-containing partners potentially targeted by ACE2 in cells. We investigated their roles and searched for potential links with ACE2 and/or SARS-CoV-2 infection.

SNX27 was the best binder of ACE2 PBM in our holdup assay. This binding was previously reported by Kliche et al. [10]. SNX27 belongs to the sorting nexin family of proteins and is involved in the recycling of internalized transmembrane proteins [19]. SNX27 is widely expressed but has been extensively studied in the context of the central nervous system (CNS) homeostasis regulation [20]. SNX27 is important for higher-order processes, such as learning and memory [21], and patients with SNX27 variants display seizures, developmental delay, behavioral disturbance and subcortical brain abnormalities [22].

SHANK1, SHANK2 and SHANK3 are targeted by the ACE2 PBM and belong to the SHANK family composed of developmental genes involved in the development and function of neural circuits, as well as in the formation of synapses. Mutations in SHANK genes involve autism spectrum disorder with altered functions in synaptic scaffolding to regulating spine morphology and neurotransmission [23].

The three members of the NHERF family, NHERF1, NHERF2 and NHERF3, are also targeted by the PBM of ACE2. The NHERF family functions are broad [24]. These scaffold proteins contain two PDZ domains and a C-terminal sequence that binds several members of the ERM (ezrin–radixin–moesin) family and they are highly expressed in epithelial tissues [25]. Many NHERF partners have been identified, broadening the protein functions in membrane physiology. NHERF is involved in transporting proteins to apical membranes and in the regulation of endocytosis and internal trafficking of membrane proteins [26]. NHERF1 was recently shown to interact through PDZ/PBM interaction with ACE2. Zhang et al. proposed that this interaction facilitates ACE2 receptor internalization and thus SARS-CoV-2 virion internalization, since it is attached through ACE2 by the viral surface spike protein [11].

Then, two members of the MAST family, MAST1 and MAST2, are targeted by the PBM of ACE2. The MAST family is composed of five genes (MAST1, MAST2, MAST3, MAST4, and MASTL) and is predominantly expressed in neuronal cells [27]. It forms a subgroup of microtubule-associated serine/threonine kinases composed of a kinase domain and a PDZ domain. They are involved in microtubule assembly, an essential step in neurogenesis. MAST1 is essential for correct brain development [28] and MAST1 substitutions are present in patients with autism and microcephaly, suggesting that mutations in this gene might be involved in neurodevelopmental diseases. MAST2 is an anti-survival kinase that interacts with PTEN in neurons [29] among other roles. MAST2 shows an association with red blood cell distribution width, which was identified recently as a biomarker of COVID-19 mortality [30]. MAST3 may play a role in developmental and epileptic encephalopathies [31].

PTPN3 is a tyrosine phosphatase, the vertebrate homolog of Ptpmeg, expressed among others in the nervous system and involved in neuronal circuit formation in the drosophila central brain [32]. PTPN3 is also often found involved in a variety of human cancers [33].

PARD3 is essential in epithelial tight junctions. It is required for the establishment of neuronal polarity and normal axon formation in cultured hippocampal neurons [34,35]. Interestingly, we previously showed that it is a potential target of the SARS-CoV-2 envelope PBM [15]. SCRIB is a polarity protein important in maintaining cell junctions and belongs to polarity complexes as PARD3.

SHROOM2 may be involved in endothelial cell morphology changes during cell spreading [36] and GRASP regulates the trafficking of metabotropic glutamate receptors that play important roles in various neuronal functions [37].

Hence, 9 out of 14 proteins have been described to play a role in neuronal cells depicting a neuronal-oriented specificity profile with a significant emphasis on cellular junctions and trafficking.

## 4. Discussion

ACE2 is involved in blood pressure regulation and cleaves a range of substrates involved in different physiological processes. ACE2 receptors, proteases such TMPRSS2, and furin are important for viral entry into host cells for coronaviruses such as SARS-CoV-2. Importantly, ACE2 is the functional receptor for SARS-CoV-2 responsible for the COVID-19 disease. ACE2 belongs to a family of transmembrane proteins that have wide tissue distribution. Protein expression of ACE2 was initially identified in the heart, kidney, and testis, and on the surface of cells that are in contact with the cellular environment such as the lung’s alveolar epithelial cells and the small intestine’s enterocytes. In addition, ACE2 is also localized in the brain [38,39].

In this work, we demonstrated that a peptide mimicking the C-terminal sequence of ACE2 containing a three-residue canonical class I PBM binds *in vitro* to several human PDZ domains. Fourteen PDZ domains (5% of the human PDZome) were identified as binders of the ACE2 PBM with Kd values ranging from 3 μM to 81 μM.

The 14 PDZ–PBM interactions found in this study may impact the SARS-CoV-2 viral infection, as recently observed for NHERF1 and SNX27. In this case, the PDZ/PBM interaction of ACE2 with NHERF1 facilitates SARS-CoV-2 internalization by enhancing the ACE2 membrane residence [11]. Interestingly, the four PDZ-containing proteins, PARD3, NHERF1, MAST2 and SNX27, were also identified as binders of the class II PBM of the envelope (E) protein of SARS-CoV-2 in our previous study [15] due to the prominent promiscuity of PDZ domains. Among these binders, we showed that PARD3 and MAST2 significantly affect viral replication under knock down gene expression in infected cells. This suggests that SARS-CoV-2 also targets ACE2 partners to potentially facilitate virus entry. NHERF, SHANK, and SNX27 proteins are involved in receptor trafficking both at the cell surface and intracellular side, which could be linked to the recycling of ACE2. SNX27 is involved in transport by the recycling of internalized transmembrane proteins [40]. It is required for SARS-CoV-2 entry [41]. Thus, SNX27 might be involved in recycling ACE2 to the plasma membrane stimulating viral entry, as previously suggested by Kliche et al. [10]. Interestingly, SNX27 was recently reported to interact with the SARS-CoV-2 spike (S) protein through its PDZ domain to facilitate S protein surface expression, although the viral protein does not contain a PBM. SARS-CoV-2 may facilitate virion trafficking to establish virus infection by this strategy [42]. Overall, among the ACE2 binders, some are known to interact with viruses such as SNX27 [43,44], MAST2, PTPN3 and SCRIB [45]. Particular attention must be paid to the cellular context because of possible different localization of the PBM-containing protein and the PDZ proteins, the level of cellular expression and the potential post-translational modifications that can affect the interaction. Future works should investigate these aspects.

The NHERF family is also a potential target of the ACE2 PBM. NHERF2 is structurally related to NHERF1. NHERF3 regulates the surface expression of plasma membrane proteins in the apical area of epithelial cells in agreement with the enrichment of ACE2 on the apical surface of airway epithelia [46]. Thus, PDZ/PBM interactions can play a role in ACE2 localization and recycling, which could be beneficial for the virus for internalization.

We found that nine PDZ-containing proteins targeted by ACE2 PBM display neuronal functions: the three SHANK, two MAST proteins, SNX27, PARD3, GRASP and PTPN3. Interestingly, the human PDZ specificity profiles we previously established for neurotropic viruses with the PBMs of the Rabies glycoprotein and of the West Nile NS5 protein mainly overlap with the PDZ profile we showed in this study [47,48]. ACE2 in the CNS is involved in the regulation of cardiovascular function and has noticeable effects on blood pressure, cardiac hypertrophy, stress response, anxiety, cognition, brain injury and neurogenesis [49,50]. ACE2 may play a role in the CNS through the PBM–PDZ interactions with the nine PDZ-containing proteins, with neuronal functions found to interact with its PBM. Interestingly, SARS-CoV-2 affects multiple organ systems, including the CNS. Most patients with COVID-19, caused by SARS-CoV-2, exhibit neurological symptoms. Neurons can be a target of SARS-CoV-2 infection, with localized ischemia in the brain and cell death, highlighting that SARS-CoV-2 neurotropism and neuronal infection can be prevented by blocking ACE2 with antibodies [51]. Indeed, the ACE2 receptor is expressed in the brain by endothelial, neuronal and glial cells. After the spreading of the virus to CNS, it comes into contact with ACE2 on neurons, glia and vascular endothelium [52]. SARS-CoV-2 might also interact directly with sensory neurons, given that sensory dysfunction, including cough, olfactory and taste impairments, are frequent in infected patients [53,54]. Recently, the loss of the cilia allowing the reception of odor molecules by sensory neurons has been reported after viral infection as well as the presence of viruses in sensory neurons. In addition, the virus invasion in the olfactory bulb as well as the presence of neuroinflammation and viral RNA in several regions of the brain have been shown [54].

Thus, we identified proteins potentially targeted by ACE2 providing new cellular protein partners through PDZ–PBM interactions. Recent proteomics of SARS-CoV-2-infected host cells did not allow us to correlate the expression of these PDZ-containing partners with the ACE2 expression profile [55]. Our study provides a resource for the research on possible biological pathways involving ACE2. Moreover, SARS-CoV-2 enters the cells through the widely expressed ACE2 receptor, affects organ systems including the CNS and it therefore has pleiotropic effects. Modifications of the interactions between ACE2 and the neuronal proteins identified in our assay could participate in the neurological alterations observed in COVID-19.

## Supplementary Materials

The following are available online at https://www.mdpi.com/article/10.3390/v13112281/s1, Table S1: All BI values obtained by holdup assay using PBM peptide from ACE2 protein. Negative BI values reflect the error and limit of detection of significant PDZ/peptide interactions as previously reported [14].
