# Supplementary material for: PDZ-Containing Proteins Targeted by the ACE2 Receptor
Source: Viruses. 2021 Nov 15;13(11):2281. doi: 10.3390/v13112281 (PMC8624105; doi:10.3390/v13112281)
Supplement: Supplementary file 1 [file viruses-13-02281-s001.zip › viruses-1331929-supplementary.pdf]

PDZ-containing proteins targeted by the ACE2 receptor

Célia Caillet-Saguy[1]* and Nicolas Wolff[1]*

* Corresponding authors. E-mails: celia.caillet-saguy@pasteur.fr and nicolas.wolff@pasteur.fr

[1] Institut Pasteur. Unité Récepteurs-Canaux. UMR CNRS 3571. 75015 Paris. France.

Table S1. All BI values obtained by holdup assay using PBM peptide from ACE2 protein.

PDZ name	BI	sd	Kd estimated
SNX27	0.87	0.03	2.57
SHANK3	0.75	0.03	6.08
MAST2	0.72	0.06	6.96
NHERF2 PDZ2	0.67	0.05	9.30
MAST1	0.63	0.10	10.71
SHANK2	0.62	0.03	11.50
PTPN3	0.54	0.06	16.24
SHANK1	0.54	0.08	16.34
NHERF3 PDZ1	0.50	0.04	19.19
NHERF1 PDZ1	0.39	0.02	31.05
PARD3	0.28	0.05	51.12
SHROOM2 PDZ1	0.26	0.04	55.74
SCRIB PDZ3	0.23	0.00	69.12
GRASP	0.20	0.01	81.33
MAST3 PDZ1	0.20	0.02	
RHPN1 PDZ1	0.16	0.06	
MAGI1 PDZ5	0.16		
NHERF2 PDZ1	0.15	0.04	
MAGI3 PDZ5	0.14	0.06	
GRID2IP PDZ2	0.14	0.21	
PTPN4 PDZ1	0.13	0.03	
DLG2 PDZ2	0.13	0.25	
GIPC1 PDZ1	0.12	0.22	
NHERF PDZ2	0.11	0.05	
FRMPD4 PDZ1	0.11	0.02	
PARD3B PDZ1	0.10	0.01	
PARD3 PDZ3	0.09	0.07	
DFNB31 PDZ1	0.09	0.02	
ARHGAP21 PDZ1	0.09	0.06	
FRMPD3 PDZ1	0.07	0.05	
MYO18A PDZ1	0.06	0.02	
MAGI1 PDZ4	0.06	0.03	
NHERF3 PDZ2	0.05	0.01	

PDLIM3 PDZ1	0.05	0.01
LIN7C PDZ1	0.05	
NHERF4 PDZ1	0.05	0.01
PDLIM1 PDZ1	0.04	0.07
MAGI1 PDZ6	0.04	
ARHGEF12 PDZ1	0.04	0.00
MPDZ PDZ9	0.03	0.13
CARD14 PDZ1	0.03	0.05
ARHGAP23 PDZ1	0.03	0.09
CNKSR2 PDZ1	0.03	0.01
CASK PDZ1	0.03	0.08
SDCBP2 PDZ1	0.03	0.07
MAGI3 PDZ3	0.03	0.05
LMO7 PDZ1	0.03	0.07
PPP1R9A PDZ1	0.02	0.00
SCRIB PDZ1	0.02	
PTPN13 PDZ4	0.02	0.15
MAGI2 PDZ6	0.02	0.03
MAGI3 PDZ1	0.02	0.07
HTRA1 PDZ1	0.02	0.06
GRIP2 PDZ2	0.02	0.02
LDB3 PDZ1	0.02	0.02
PSMD9 PDZ1	0.02	0.01
InaDl PDZ5	0.02	0.04
TJP1 PDZ2	0.01	0.02
PSCDBP PDZ1	0.01	0.04
LNX2 PDZ4	0.01	0.06
PDZD11 PDZ1	0.01	0.02
PPP1R9B PDZ1	0.01	0.01
CARD11 PDZ1	0.01	0.03
DVL3 PDZ1	0.01	0.08
PDZRN3 PDZ2	0.01	
InaDl PDZ9	0.01	0.04
AHNAK1 PDZ1	0.00	
CNKSR3 PDZ1	0.00	
SIPA1L1 PDZ1	0.00	0.03
MPDZ PDZ7	0.00	0.02
RADIL PDZ1	0.00	0.06
PDZD7 PDZ1	0.00	0.06
MAST4 PDZ1	0.00	0.04
GRIP2 PDZ5	0.00	0.03
SNTG1 PDZ1	-0.01	0.02
PDZD2 PDZ4	-0.01	0.01

InaDl PDZ2	-0.01	0.01
GRIP1 PDZ3	-0.01	
DEPTOR PDZ1	-0.01	
InaDl PDZ7	-0.01	0.08
MPDZ PDZ4	-0.01	0.02
DLG5 PDZ4	-0.01	0.04
LNX2 PDZ2	-0.01	0.02
GRIP1 PDZ1	-0.01	0.10
SNTB1 PDZ1	-0.01	0.10
InaDl PDZ4	-0.01	0.04
PDZRN3 PDZ1	-0.01	0.13
PARD3B PDZ3	-0.01	0.02
LIN7B PDZ1	-0.01	0.05
SDCBP2 PDZ2	-0.01	0.04
DLG2 PDZ1	-0.01	0.02
NHERF3 PDZ3	-0.01	0.02
TJP1 PDZ1	-0.01	0.05
MLLT4 PDZ1	-0.01	0.01
DLG3 PDZ3	-0.02	0.01
LAP2 PDZ1	-0.02	0.09
RIMS2 PDZ1	-0.02	0.11
RGS12 PDZ1	-0.02	0.03
DLG4 PDZ2	-0.02	0.01
SCRIB PDZ4	-0.02	0.02
PARD3 PDZ2	-0.02	0.02
SHROOM4 PDZ1	-0.02	0.07
PTPN13 PDZ3	-0.02	0.02
MPP4 PDZ1	-0.02	0.04
PTPN13 PDZ1	-0.02	0.02
NHERF4 PDZ3	-0.02	0.03
RIMS1 PDZ1	-0.02	0.07
PDZRN4 PDZ2	-0.02	0.00
NOS1 PDZ1	-0.02	0.00
FRMPD2 PDZ1	-0.02	0.05
LNX1 PDZ4	-0.02	0.06
PDZD9 PDZ1	-0.02	0.11
GIPC3 PDZ1	-0.02	0.05
DLG5 PDZ2	-0.03	0.02
STXB4 PDZ1	-0.03	0.07
GRID2IP PDZ1	-0.03	0.02
SIPA1L2 PDZ1	-0.03	0.00
SCRIB PDZ2	-0.03	0.06
PRX PDZ1	-0.03	0.01

PDZD2 PDZ2	-0.03	0.02
FRMPD1 PDZ1	-0.03	0.03
InaDl PDZ6	-0.03	0.00
MAGI1 PDZ1	-0.03	0.06
PDZD7 PDZ2	-0.03	0.03
PDZD2 PDZ3	-0.03	0.02
MPP5 PDZ1	-0.03	0.01
SNTA1 PDZ1	-0.03	0.03
MAGI2 PDZ2	-0.03	0.01
PDZD2 PDZ6	-0.03	0.05
PICK1 PDZ1	-0.03	
DLG4 PDZ3	-0.03	0.05
APBA2 PDZ1	-0.03	
FRMPD2 PDZ3	-0.03	0.03
GORASP2 PDZ1	-0.03	0.04
MAGI3 PDZ6	-0.03	0.01
MPDZ PDZ6	-0.03	0.03
MPDZ PDZ13	-0.03	0.03
MAGI1 PDZ2	-0.04	0.00
APBA1 PDZ2	-0.04	
MAGI2 PDZ5	-0.04	0.02
RGS3 PDZ1	-0.04	0.07
DLG3 PDZ1	-0.04	0.06
PDLIM4 PDZ1	-0.04	0.00
MAGI2 PDZ1	-0.04	0.01
MAGI1 PDZ3	-0.04	0.01
MPP3 PDZ1	-0.04	0.02
IL16 PDZ3	-0.04	0.01
GRIP2 PDZ7	-0.04	0.06
AHNAK2 PDZ1	-0.04	
SIPA1 PDZ1	-0.04	0.01
MAGI2 PDZ4	-0.04	0.01
LIN7A PDZ1	-0.04	0.05
PDZD8 PDZ1	-0.04	0.00
DLG2 PDZ3	-0.04	0.05
GRIP2 PDZ3	-0.04	0.00
MPP7 PDZ1	-0.04	0.00
GRIP2 PDZ6	-0.04	0.02
GRIP1 PDZ7	-0.04	0.02
SHROOM3 PDZ1	-0.04	0.03
MPDZ PDZ3	-0.04	0.07
GIPC2 PDZ1	-0.05	0.04
PREX2 PDZ2	-0.05	0.01

MPDZ PDZ2	-0.05	0.03
PREX1 PDZ2	-0.05	0.00
MAGI3 PDZ2	-0.05	0.00
PDZRN4 PDZ1	-0.05	0.06
MPDZ PDZ1 2	-0.05	0.01
PCLO PDZ1	-0.05	0.01
MPP6 PDZ1	-0.05	0.00
PDLIM7 PDZ1	-0.05	0.00
SYNPO2L PDZ1	-0.05	0.04
PTPN13 PDZ5	-0.05	0.04
SNTG2 PDZ1	-0.05	0.02
SYNP2 PDZ1	-0.05	0.02
MPDZ PDZ11	-0.05	0.04
HTRA4 PDZ1	-0.05	0.01
MAGI3 PDZ4	-0.05	0.01
DFNB31 PDZ2	-0.05	0.02
PDLIM2 PDZ1	-0.05	0.00
SIPA1L3 PDZ1	-0.05	0.02
MPDZ PDZ5	-0.05	0.04
PDZD4 PDZ1	-0.05	0.02
PARD6G PDZ1	-0.05	0.00
MPDZ PDZ8	-0.06	0.03
HTRA3 PDZ1	-0.06	0.00
IL16 PDZ1	-0.06	0.03
PDZD7 PDZ3	-0.06	0.05
RAPGEF2 PDZ1	-0.06	
MAGI2 PDZ3	-0.06	0.01
LRRC7 PDZ1	-0.06	0.01
GORASP1 PDZ1	-0.06	0.06
DLG5 PDZ3	-0.06	0.08
SYNJ2BP PDZ1	-0.06	0.02
InaDl PDZ3	-0.06	0.05
APBA2 PDZ2	-0.06	0.01
RAPGEF6 PDZ1	-0.06	0.02
NHERF3 PDZ4	-0.06	0.19
MAGIX PDZ1	-0.06	0.01
NHERF4 PDZ2	-0.06	0.10
LNX1 PDZ1	-0.07	0.04
DLG1 PDZ2	-0.07	0.03
MPDZ PDZ1 0	-0.07	0.01
LNX1 PDZ2	-0.07	
PARD3B PDZ2	-0.07	0.06
LNX1 PDZ3	-0.07	0.03

TJP2 PDZ1	-0.07	0.03
GRIP1 PDZ2	-0.07	0.02
GRIP1 PDZ5	-0.07	0.03
LNX2 PDZ1	-0.07	0.01
INTU PDZ1	-0.07	0.05
MPDZ PDZ1	-0.07	0.01
MPP1 PDZ1	-0.07	0.05
FRMPD2 PDZ2	-0.07	0.03
DLG1 PDZ3	-0.07	0.02
PDLIM5 PDZ1	-0.08	0.02
SDCBP PDZ1	-0.08	0.02
HTRA2 PDZ1	-0.08	0.01
TIAM1 PDZ1	-0.08	0.04
APBA1 PDZ1	-0.08	
RHPN2 PDZ1	-0.08	0.04
TJP3 PDZ2	-0.08	0.08
GRIP2 PDZ4	-0.08	0.09
SNTB2 PDZ1	-0.08	0.07
PARD6A PDZ1	-0.08	0.00
TJP3 PDZ3	-0.08	0.01
SDCBP PDZ2	-0.09	0.04
InaDl PDZ10	-0.09	0.04
IL16 PDZ4	-0.09	0.00
PTPN13 PDZ2	-0.09	0.10
GOPC PDZ1	-0.09	0.02
TX1B3 PDZ1	-0.09	0.04
PDZD2 PDZ1	-0.09	0.02
IL16 PDZ2	-0.09	0.03
APBA3 PDZ2	-0.10	
LIMK1 PDZ1	-0.10	0.08
GRIP1 PDZ4	-0.10	0.06
DLG5 PDZ1	-0.10	
PREX1 PDZ1	-0.10	0.05
PARD6B PDZ1	-0.10	0.01
TJP3 PDZ1	-0.10	0.02
TJP2 PDZ3	-0.10	
APBA3 PDZ1	-0.11	0.18
LIMK2 PDZ1	-0.11	0.03
GRIP2 PDZ1	-0.11	0.13
USH1C PDZ3	-0.11	0.01
PREX2 PDZ1	-0.11	0.06
NHERF4 PDZ4	-0.11	0.08
MPP2 PDZ1	-0.12	0.02

TJP2 PDZ2	-0.12	0.04
TIAM2 PDZ1	-0.12	0.13
PDZD2 PDZ5	-0.13	0.02
USH1C PDZ1	-0.13	
DVL1 PDZ1	-0.14	0.18
DLG1 PDZ1	-0.14	0.19
TJP1 PDZ3	-0.14	0.10
ARHGEF11 PDZ1	-0.18	0.19
InaDl PDZ8	-0.24	0.29
DFNB31 PDZ3	-0.24	0.33
USH1C PDZ2	-0.25	0.10
DLG3 PDZ2	-0.27	0.38
GRIP1 PDZ6	-0.27	0.36
CNKSR1 PDZ1	-0.31	0.38
InaDl PDZ1	-0.34	0.31
DVL2 PDZ1	-0.38	0.57
DLG4 PDZ1	-0.50	
LNX2 PDZ3		
